# Epidemiological patterns of syndromic symptoms in suspected patients with COVID-19 in Iran: A Latent Class Analysis

**DOI:** 10.34172/jrhs.2021.41

**Published:** 2021-01-18

**Authors:** Ali Hosseinzadeh, Maysam Rezapour, Marzieh Rohani-Rasaf, Mohammad Hassan Emamian, Seyedeh Solmaz Talebi, Shahrbanoo Goli, Reza Chaman, Hossein Sheibani, Ehsan Binesh, Fariba Zare, Ahmad Khosravi

**Affiliations:** ^1^Department of Epidemiology, School of Public Health, Shahroud University of Medical Sciences, Shahroud, Iran; ^2^Amol Faculty of Paramedical Sciences, Mazandaran University of Medical Sciences, Sari, Iran; ^3^Ophthalmic Epidemiology Research Center, Shahroud University of Medical Sciences, Shahroud, Iran; ^4^Clinical Research Development Unit, Imam Hossein Hospital, Shahroud University of Medical Sciences, Shahroud, Iran; ^5^Center for Health-Related Social and Behavioral Sciences Research, Shahroud University of Medical Sciences, Shahroud, Iran

**Keywords:** COVID-19, Latent Class Analysis, Epidemiological pattern, Diagnosis

## Abstract

**Background:** Early diagnosis and supportive treatments are essential to patients with coronavirus disease 2019 (COVID-19). Therefore, the current study aimed to determine different patterns of syndromic symptoms and sensitivity and specificity of each of them in the diagnosis of COVID-19 in suspected patients.

**Study Design:** Cross-sectional study

**Methods:** In this study, the retrospective data of 1,539 patients suspected of COVID-19 were obtained from a local registry under the supervision of the officials at Shahroud University of Medical Sciences, Shahroud, Iran. A Latent Class Analysis (LCA) was carried out on syndromic symptoms, and the associations of some risk factors and latent subclasses were accessed using one-way analysis of variance and Chi-square test.

**Results:** The LCA indicated that there were three distinct subclasses of syndromic symptoms among the COVID-19 suspected patients. The age, former smoking status, and body mass index were associated with the categorization of individuals into different subclasses. In addition, the sensitivity and specificity of class 2 (labeled as "High probability of polymerase chain reaction [PCR]^+^ ") in the diagnosis of COVID-19 were 67.43% and 76.17%, respectively. Furthermore, the sensitivity and specificity of class 3 (labeled as "Moderate probability of PCR^+^ ") in the diagnosis of COVID-19 were 75.92% and 50.23%, respectively.

**Conclusions:** The findings of the present study showed that syndromic symptoms, such as dry cough, dyspnea, myalgia, fatigue, and anorexia, might be helpful in the diagnosis of suspected COVID-19 patients.

## Introduction

Coronavirus disease 2019 (COVID-19) is a coronavirus outbreak that was initially identified in Wuhan, China, in late 2019 ^1^. The typical symptoms are fever, dry cough, fatigue, dyspnea, and loss of smell and taste ^2,3^. This infection has now spread worldwide and caused more than 4.89 million cases and more than 323,000 mortalities across 188 countries by May 20, 2020 ^4^. Based on the World Health Organization declaration, COVID-19 has become a global health concern ^2,5^.

Currently, controlling the COVID-19 epidemic is one of the top priorities of all countries around the world. To detect this novel coronavirus, molecular-based approaches, including the reverse transcription-polymerase chain reaction (RT-PCR), are the first methods for the confirmation of suspected cases ^6,7^. The RT-PCR test is usually performed on respiratory specimens obtained from a nasopharyngeal swab; however, a nasal swab or sputum specimen may also be used ^7^. In Iran, as in other countries of the world, the RT-PCR test is utilized to confirm COVID-19 in suspected patients. The current laboratory tests are time-consuming and there is a shortage of commercial kits in many geographical areas and developing countries leading to delay in the diagnosis of the infection ^2,8^. The rapid and accurate detection of COVID-19 is crucial to control outbreaks in communities and hospitals ^9^. Therefore, the use of methods that are available everywhere and are not expensive seems necessary for the diagnosis of COVID-19 in its early stage.

 One of the most cost-effective diagnostic methods for the diagnosis of COVID-19, which is available in all geographical areas, is the identification of the disease symptoms. The patterns of syndromic symptoms can be extracted by Latent Class Analysis (LCA), which is a statistical method for the recognition of the subtypes of related cases (i.e., latent classes) using multivariate categorical data. In LCA, a person-oriented grouping approach is used, which can simultaneously consider the effects of many contexts (e.g., race, genetics, society, and environment) on the classification of individuals as latent clusters that are homogeneous. Therefore, the current study aimed to determine different patterns of syndromic symptoms and sensitivity and specificity of each of them in the diagnosis of COVID-19 in suspected patients.

## Methods

###  Source of data 

With the onset of the COVID-19 epidemic, a local registration system was established in Shahroud, Iran, under the supervision of Shahroud University of Medical Sciences. According to the recommendation of the National Health Commission, the RT-PCR test was used for all suspected patients and those who were in contact with them in Shahroud within December 11, 2019, and January 29, 2020. In the present study, a confirmed case of COVID-19 was defined as the one in which the RT-PCR test was positive based on a nasal or nasopharyngeal swab. For all the suspected patients, an electronic medical record was created, including demographic variables (e.g., age, gender, height, weight, and place of residence) and some other variables (e.g., used sampling method, patient comorbidities, smoking history, medicinal use history, medical history, history of cardiac monitoring, oxygen therapy, radiological assessment [e.g., chest X-ray or computed tomography scan], and other taken measures necessary to care for the patient). Therefore, the collected data seem to be relatively comprehensive, especially in individuals of over 60 years of age. However, the number of patients who have not been diagnosed and registered may remain underestimated. The current study was conducted based on the data retrieved from this COVID-19 registry in the interval of February 20 and May 8, 2020. It is noteworthy that in this study, the expectation-maximization algorithm was used for the estimation of missing data. This algorithm is considered by some researchers as the most accurate method to estimate missing data ^10^.

###  Data analysis

 The analysis was based on the objectives of the study and carried out in three steps. In the first step, LCA was employed to extract the patterns of COVID-19 symptoms. The LCA is a model-based person-oriented approach to categorize similar individuals into groups according to primitive symptoms. The LCA was performed in an exploratory manner starting from a two-class model and continuing up to a five-class model. The five-class model failed to converge; therefore the latent class models were compared in order to decide what the optimal number of classes would be.

Model fit statistics in combination with empirical evidence and interpretability were used to determine the optimal number of classes. These different statistical indices included the Akaike information criterion (AIC), Bayesian information criterion (BIC), and sample-size adjusted Bayesian information criterion (aBIC). Smaller values of the AIC, BIC, and aBIC indicated a model with a better fit ^11,12^. In addition, the Lo-Mendell-Rubin likelihood ratio test (LMR-LRT), Vuong-Lo-Mendell-Rubin likelihood ratio test (VLMR-LRT), and parametric bootstrap likelihood ratio test were used. The significant p-value of these tests favored the k class model over the k-1 class model ^13^. Entropy values within the range of 0-1 were also utilized. A higher value of entropy indicates a model with a better fit, and there is a clear separation of classes in values above 80 ^14^.

 In the second step, one-way analysis of variance (ANOVA), Tukey’s posthoc test, and Chi-square tests were applied for the examination of the associations between the identified patterns (i.e., classes) and independent variables.

Finally, in the third step, the diagnostic accuracy of the extracted classes of COVID-19 symptoms was evaluated in terms of sensitivity, specificity, positive predictive value (PPV), and negative predictive value (NPV) using RT-PCR as the gold standard. The data management, descriptive analyses, one-way ANOVA, Chi-square test, and diagnostic accuracy evaluations were carried out in Stata software (version 14). Moreover, LCA was carried out in Mplus software (version 7.4) ^15^.

## Results

Up to May 8, 2020, 1,539 suspected patients (808 females [52.8%]) were tested, out of whom the RT-PCR test was positive for 529 cases (34.4%). The mean age values in individuals with and without COVID-19 were 56.60±17.51 and 49.99±18.41 years, respectively (P<0.001). The mean score of body mass index (BMI) for individuals with and without COVID-19 were 27.12±4.35 and 26.44±4.16, respectively (P<0.001). The most common symptoms were dry cough (47.3%), anorexia (41.1%), dyspnea (39.3%), and fever (38.6%) (Table 1).

**Table 1:** Symptoms observed in suspected patients

**Item**	**Number**	**Percent**
Fever	594	38.6
Sore throat	318	20.7
Dry cough	727	47.3
Dyspnea	605	39.3
Runny nose	80	5.2
Trembling	455	29.6
Vomiting	174	11.3
Nausea	298	19.4
Diarrhea	183	11.9
Headache	473	30.8
Myalgia	499	32.4
Arthralgia	338	22.0
Anorexia	631	41.0
Fatigue	561	36.5
Loss of consciousness	74	4.8

A series of two to six latent class models were estimated based on the 15 primitive symptoms (Table 2). The AIC of the four- and five-class models had lower values of AIC, BIC, and aBIC than those reported for the three-class model. However, the VLMR-LRT and LMR-LRT were significant in two- and three-class models and not significant in four- and five-class models. As a result, the model with the best fit was hard to identify. However, with regard to the entropy value, parsimony, and interpretability, the three-class model was preferred to other models ^16^.

**Table 2:** Fit indices of Latent Class Analysis

**Variable**	**AIC**	**BIC**	**aBIC**	**VLMR-LRT**	**LMR-LRT**	**BLRT**	**Entropy**
Class 2	22353.95	22519.44	22420.96	1884.4^b^	1868.5^b^	1884.4^b^	0.75
Class 3	22093.53	22344.43	22195.12	292.40^a^	289.90^a^	292.40^b^	0.71
Class 4	21923.90	22260.21	22060.07	201.63	199.92	201.63	0.68
Class 5	21831.68	22253.41	22002.44	124.21	123.16	124.21	0.71

AIC: Akaike information criterion; BIC: Bayesian information criterion; aBIC: Adjusted Bayesian information criterion; VLMR-LRT: Vuong-Lo-Mendell-Rubin likelihood ratio; LMR-LRT : Lo-Mendell-Rubin likelihood ratio test; BLRT: Bootstrap likelihood ratio test ^a^ P<0.050 b^b^P<0.001

**Figure 1:**
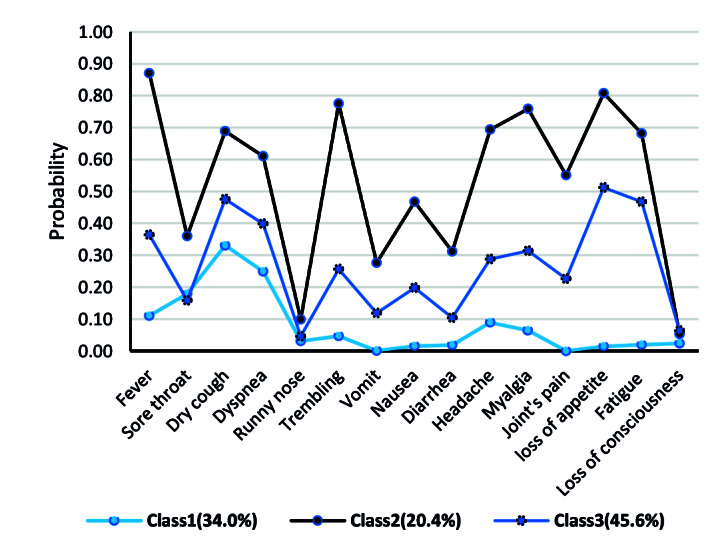
Illustration of probabilities of symptoms in each class

Figure 1 depicts a visual inspection of the solution with three class models, where each primitive symptom is plotted on the *x*-axis and the symptom-class probabilities are plotted on the *y*-axis. Latent class 1 (labeled as “Low probability of PCR^+^”), with a prevalence of 34.0% (n=523), was characterized by a low probability of all symptoms for individuals clustered in this class (P<0.300). Latent class 2 (labeled as “High probability of PCR^+^”), with a prevalence of 20.4% (n=313), was characterized by a high probability (P>0.600) of fever, dry cough, dyspnea, trembling, headache, myalgia, arthralgia, anorexia, and fatigue for individuals clustered in this class. Latent class 3 (labeled as “Moderate probability of PCR^+^”), with a prevalence of 45.6% (n=702), was characterized by a medium probability (P=0.300 to 0.500) of fever, dry cough, dyspnea, myalgia, anorexia, and fatigue for individuals clustered in this class.

Table 3 tabulates the associations of the latent classes with age, BMI, gender, marital status, former smoking status, current smoking status, ethnicity, flu during the last year, and history of flu vaccination. The age and BMI varied across the three latent classes (F=34.7 and *P*<0.001 for age; F=11.9 and *P*<0.001 for BMI). Classes 3 and 1 had the highest and lowest mean age, respectively. Classes 2 and 1 had reported with the highest and lowest mean BMI, respectively. The former smoking status was a significant variable for the variations of the latent classes (χ^2^=9.19; *P*<0.012). Having the flu in the last year was significant for the variations of the latent classes (χ^2^=11.61; *P*<0.011).

To calculate the sensitivity, specificity, PPV, and NPV of different classes of the symptoms, 2 × 2 contingency tables were used for each comparison. Class 1 had a very low probability of all the symptoms; therefore, it was labeled as a class with no symptoms and considered a reference group. Classes 2 and 3 were compared to class 1. The sensitivity and specificity of class 2 were 67.43% and 76.17%, respectively. In addition, the sensitivity and specificity of class 3 were 75.92% and 50.23%, respectively (Table 4).

**Table 3:** Means values, frequency, and percentage of related variables based on latent profile membership

**Characteristics**	**Class 1**		**Class 2**		**Class 3**		**F-statistics**	***P*** **-value**
**Continuous variable s**	**Mean**	**SD**	**Mean**	**SD**	**Mean**	**SD**		
Age (year)	47.27	18.97	52.52	15.91	55.53	18.11	34.65	0.003
BMI	26.07	4.08	27.52	4.42	26.75	4.20	11.88	0.282
Education (year)	9.42	5.92	9.05	7.72	8.30	7.75	3.81	0.022
**Categorical variable s**	**Number**	**Percent**	**Number**	**Percent**	**Number**	**Percent**	**χ** ^2^ **-statistics**	***P*** **-value**
PCR+^+^patients	85	16.1	176	33.3	268	50.6	146.8	0.001
Gender								
Male	276	34.2	159	19.7	373	46.2	0.49	0.782
Female	247	33.8	154	21.1	329	45.1		
Former smoker								
Yes	73	38.8	23	12.2	92	48.9	9.19	0.010
No	445	33.2	290	21.7	604	45.1		
Current smoker								
Yes	43	43.0	14	14.0	43	43.0	4.94	0.084
No	475	33.3	299	21.0	652	45.7		
Ethnicity								
Fars	519	34.2	308	20.3	692	45.6	2.23	0.327
Others	3	17.6	5	29.4	9	52.9		
Getting the flu last year								
Yes	76	27.5	76	27.5	124	44.9	11.91	0.002
No	438	35.1	237	19.0	573	45.9		
Flu vaccination history								
Yes	61	30.0	48	23.6	94	46.3	2.03	0.362
No	449	34.1	263	20.0	603	45.9		

SD: Standard deviation; BMI: Body mass index; PCR: Polymerase chain reaction Class 1: Low probability of PCR^+^ Class 2: High probability of PCR^+^ Class 3: Moderate probability of PCR^+^

**Table 4:** Sensitivity and specificity according to classes

**Class**	**%Sensitivity** **(95% CI)**	**%Specificity** **(95% CI)**	**%Positive predictive value** **(95% CI)**	**%Negative predictive value (95% CI)**	**%Accuracy** **(95% CI)**
Class 2	67.43 (61.38, 73.08)	76.17 (72.48, 79.60)	56.23 (52.04, 60.33)	83.74 (81.14, 86.06)	73.44 (70.31, 76.41)
Class 3	75.92 (71.11, 80.29)	50.23 (46.86, 53.60)	38.17 (36.10, 40.29)	83.74 (80.90, 86.25)	57.63 (54.81, 60.42)

Class 2: High probability of PCR^+^ Class 3: Moderate probability of PCR

## Discussion

The results of the current study showed that the symptoms, including sore throat, runny nose, vomiting, nausea, diarrhea, and loss of consciousness, were not informative in the syndromic diagnosis in suspected patients of COVID-19. However, dry cough, dyspnea, myalgia, fatigue, and anorexia were informative in such diagnoses. Accordingly, the sensitivity and specificity of class 2, including the aforementioned symptoms, in the diagnosis of the suspected patients of COVID-19 was relatively good. Based on the report of Center for Disease Control and Prevention (CDC) and the results of Huang et al.'s study ^17,18^, the most commonly reported symptoms are fever, cough, myalgia or fatigue, pneumonia, and shortness of breath. The less-common reported symptoms include headache, diarrhea, hemoptysis, runny nose, and phlegm-producing cough. Therefore, it seems that class 2 can be used for the screening of COVID-19 suspected patients in the primary health-care services centers. Furthermore, suspected patients with the aforementioned symptoms should be seriously regarded as COVID-19 patients in the primary levels.

Consistent with the findings of the present study, the results of other studies have suggested that the symptoms of fever, cough, and shortness of breath can be used in the early diagnose of COVID-19 suspected patients ^19,20^. However, it should be noted that the use of these symptoms for screening suspected patients may increase the risk of omitting those patients with other symptoms and a normal body temperature ^20^. Therefore, caution should be exercised when dealing with patients who are suspected of having COVID-19 but with a normal body temperature and visiting various outpatient clinics for nonrespiratory symptoms.

In the current analysis, former smoking status contributed to distinguishing patient subclasses indicating that former smoking in populations might be related to COVID-19. Although comparisons to other results are difficult given the limited available data, the findings of the current study are consistent with the reported results of Zhang et al. ^21^. In the aforementioned study, 3.4% and 6.9% of severe patients were current and former smokers, respectively, leading to an odds ratio of 2.23. In addition, a recent systematic review carried out by Vardavas and Nikitara concluded that smoking is most likely associated with negative progression and adverse outcomes of COVID19 ^22^.

However, in the present study, active smoking did not contribute to categorizing individuals into different subclasses in LCA. This finding is in line with the results of a study conducted by Lippi and Henry, demonstrating no apparent association of active smoking with enhanced risk of progression to severe disease ^23,24^. This may potentially be due to a lower level of angiotensin‐converting enzyme 2 in smokers ^24^; nevertheless, there has not currently been any solid explanation for this finding. Therefore, it is required to carry out well-designed population-based studies in order to address this issue.

In the present study, BMI was associated with a chance of membership in all subclasses, indicating that BMI might be related to COVID-19. Such a relationship has been reported in other studies. For example, in a study conducted by Radwan Kassir, BMI was significantly higher in those with severe COVID-19, compared to that reported for normal patients ^25^. Similarly, the results of studies carried out by Luca Busetto, Norbert Stefan, and Nick Finer demonstrated a relationship between obesity and COVID-19 ^26-28^. It should be noted that experiment and genetic evidence conclusively show that obesity is causally related to hypertension, diabetes, coronary heart disease, stroke, atrial fibrillation, renal disease, and heart failure ^29^. Therefore, the association between BMI and COVID-19 may be related to obesity complications, and caution should be exercised in the interpretation of this relationship.

 One of the most important limitations of the current study was no access to the data on all the symptoms associated with COVID-19. Therefore, it is suggested to include other key symptoms, such as loss of smell and taste, in future investigations. Another limitation of this study may be limited access to all suspected patients due to a lack of RT-PCR testing, especially in individuals under 60 years of age. However, it was tried to identify all suspicious patients based on contact tracing.

## Conclusions

 The findings of the present study showed that syndromic symptoms, such as dry cough, dyspnea, myalgia, fatigue, and anorexia, are helpful in the diagnosis of suspected COVID-19 patients. Therefore, syndromic symptoms can be used for diagnosis, especially at the primary health-care services centers.

## Acknowledgements

 The present study was supported by Shahroud University of Medical Sciences (grant no.: 98126).

## Conflict of interest

 The authors declare that there is no conflict of interest.

## Funding

 The current study was supported by Shahroud University of Medical Sciences (grant no.: 98126)

## Authors ʼ contributions

 Data analysis and interpretation were performed by AH and MR. AH wrote the first draft of the manuscript and it was critically revised by MHE, RCH, MR, ST, SHG, HS, EB, FZ, and AKH. All the authors read and approved the final version of the manuscript and agreed to be responsible for all the aspects of the study.

## Ethical approval

 The current study was approved by the Ethics Council of Shahroud University of Medical Sciences (IR.SHMU.REC.1398.160).

## Highlights

- There are three distinct subclasses of syndromic symptoms among coronavirus disease 2019 (COVID-19) suspected patients.
- The syndromic symptoms, such as dry cough, dyspnea, myalgia, fatigue, and anorexia, might be helpful in the diagnosis of COVID-19 suspected patients.
- The sensitivity and specificity of class 2, including dry cough, dyspnea, myalgia, fatigue, and anorexia, in the diagnosis of COVID-19 were 67.43% and 76.17%, respectively.
